# Supplementary material for: Chloroplast Cell-Free Systems from Different Plant Species as a Rapid Prototyping Platform
Source: ACS Synth Biol. 2024 Jul 19;13(8):2412–24. doi: 10.1021/acssynbio.4c00117 (PMC11334176; doi:10.1021/acssynbio.4c00117)
Supplement: Supplementary file 1 — sb4c00117_si_001.pdf [file sb4c00117_si_001.pdf]

Supporting Information for

Chloroplast Cell-Free Systems from Different Plant Species as a Rapid Prototyping Platform

Clemens V. Böhm[1,2#] & René Inckemann[1,2#*], Michael Burgis[2], Jessica Baumann[3], Cedric K. Brinkmann[1], Katarzyna E. Lipinska[1,2], Sara Gilles[1,2], Jonas Freudigmann[3], Vinca N. Seiler[3], Lauren G. Clark[4], Michael C. Jewett[4], Lars M. Voll[2,3*], Henrike Niederholtmeyer[1,2,5*]

[1] Max-Planck Institute for Terrestrial Microbiology, 35043 Marburg, Germany
[2] Center for Synthetic Microbiology, Philipps-Universität Marburg, 35032 Marburg, Germany
[3] Department of Biology, Philipps-Universität Marburg, 35043 Marburg, Germany
[4] Department Of Chemical and Biological Engineering, Northwestern University, Evanston, Illinois 60208, United States
[5] Technical University of Munich, Campus Straubing for Biotechnology and Sustainability, 94315 Straubing, Germany

[#]Contributed equally
*Corresponding authors.

Contact information for corresponding authors

René Inckemann - Max-Planck Institute for Terrestrial Microbiology, Marburg 35043, Germany; Center for Synthetic Microbiology, Philipps-Universität Marburg, Marburg 35032, Germany;
Orcid: https://orcid.org/0000-0002-6221-6443;
Email: rene.inckemann@mpi-marburg.mpg.de

Lars M. Voll - Philipps-Universität Marburg, Department of Biology, Molecular Plant Physiology, Marburg 35043, Germany; Center for Synthetic Microbiology, Philipps-Universität Marburg, Marburg 35032, Germany
Orcid: https://orcid.org/0000-0002-8723-9131
Email: lars.voll@biologie.uni-marburg.de

Henrike Niederholtmeyer - Technical University of Munich, Campus Straubing for Biotechnology and Sustainability, Straubing 94315, Germany; Max-Planck Institute for Terrestrial Microbiology, Marburg 35043, Germany; Center for Synthetic Microbiology, Philipps-Universität Marburg, Marburg 35032, Germany
Orcid: https://orcid.org/0000-0002-1375-0287
Email: henrike.niederholtmeyer@tum.de

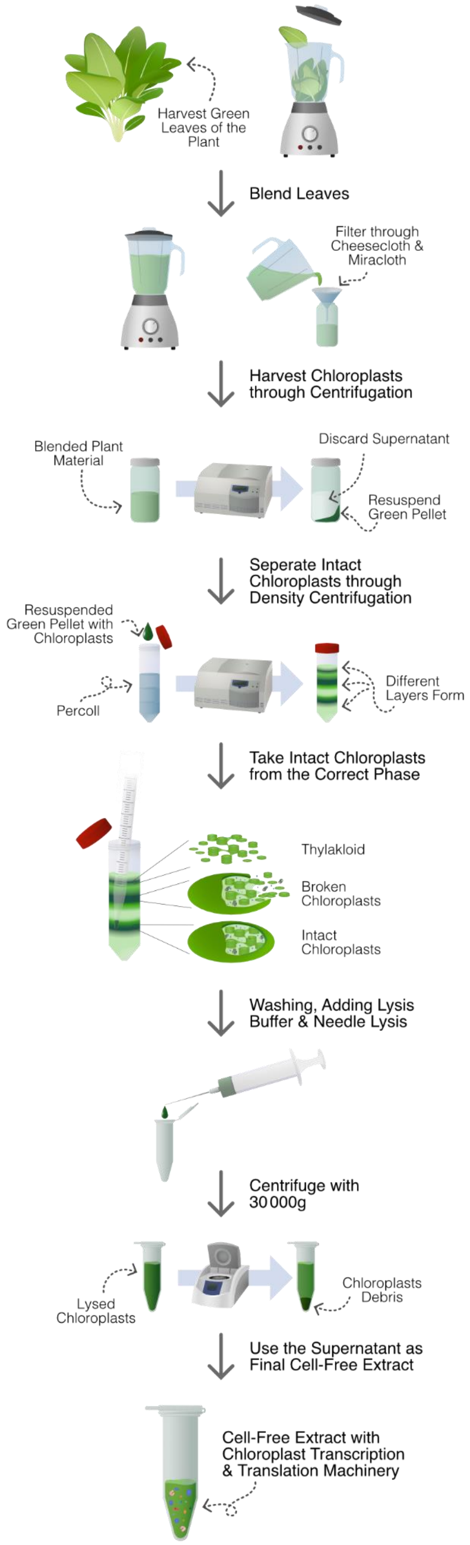

Supplementary Figure S1: **Workflow of chloroplast isolation.** Plant leaves were harvested and homogenized using a blender. After cloth filtering, the homogenate was centrifuged and the pellet resuspended. Percoll gradients were used to separate intact chloroplasts from broken ones. After washing, chloroplasts were lysed using a needle. Chloroplast debris was removed via ultracentrifugation and supernatant used as final cell-free extract.

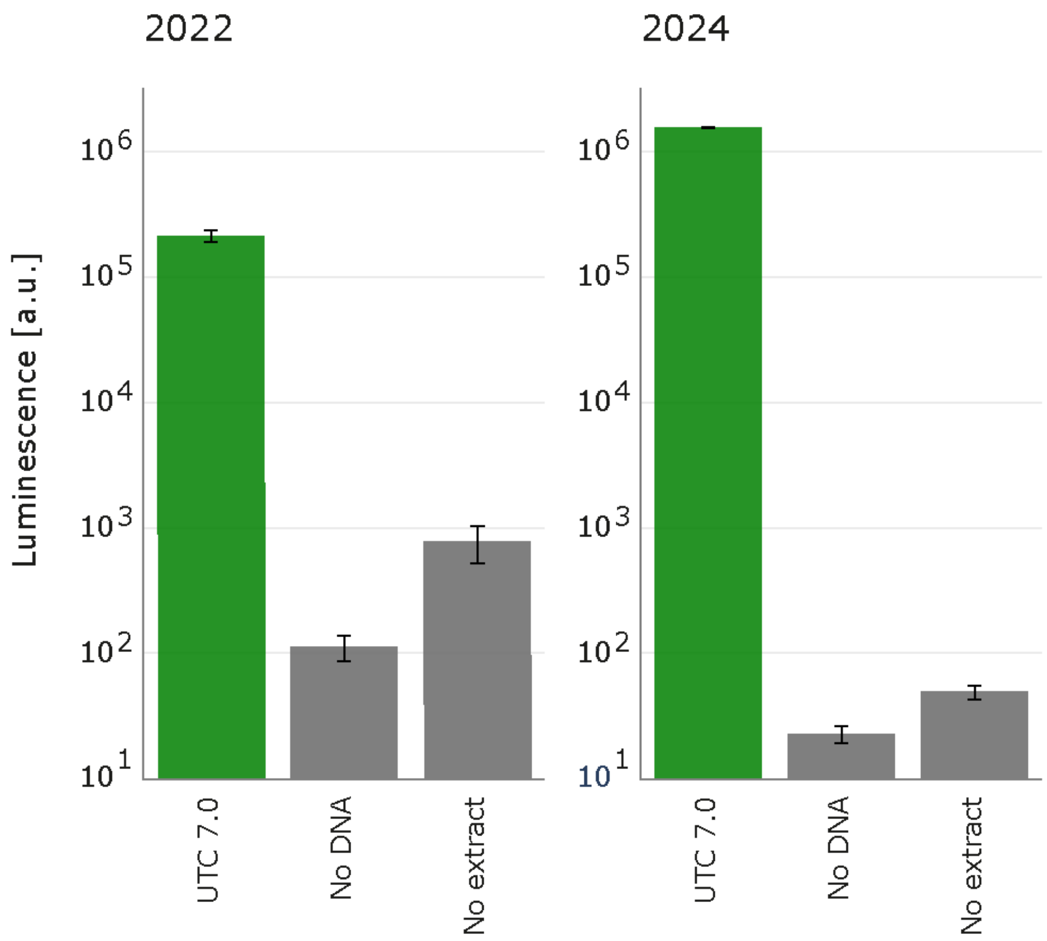

Figure S2: Chloroplast extract remains stable in storage at -80°C. The same spinach CFE batch, frozen in aliquots at -80°C, was tested in experiments that were 17 months apart. The higher luminescence signals in the 2024 experiment can be explained by a new translation buffer batch. Cell-free reactions were set up with a total volume of 2 µl and the UTC 7.0 plasmid. Luminescence was measured after 4 hours of incubation at 20°C (N=5).

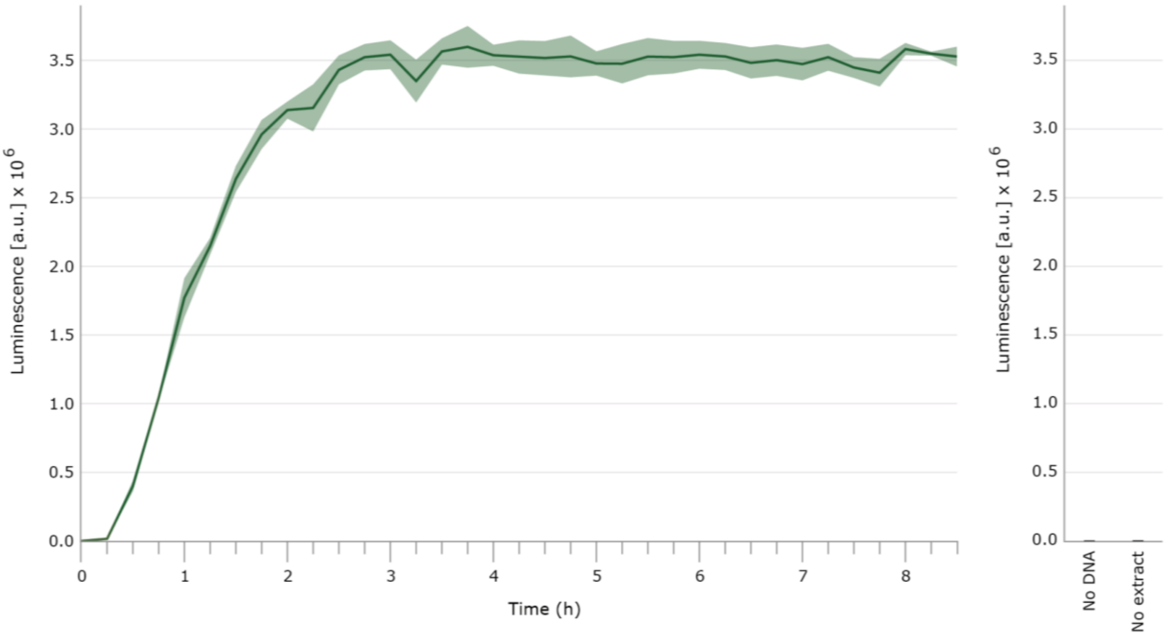

Figure S3: Kinetic of a cell-free reaction shows stable signal after 3 hours reaction time. 10 µl samples were taken every 15 minutes and frozen in liquid nitrogen for storage. Directly upon thawing, Nano-Glo assay reagents were added and finally all samples were measured simultaneously. NanoLuc luminescence signals were obtained in reactions using spinach chloroplast extract and 10 nM universal test construct plasmid DNA. Lightly shaded regions indicate standard deviations. Negative controls measured after 8.5 hours lack either DNA or extract (right panel).

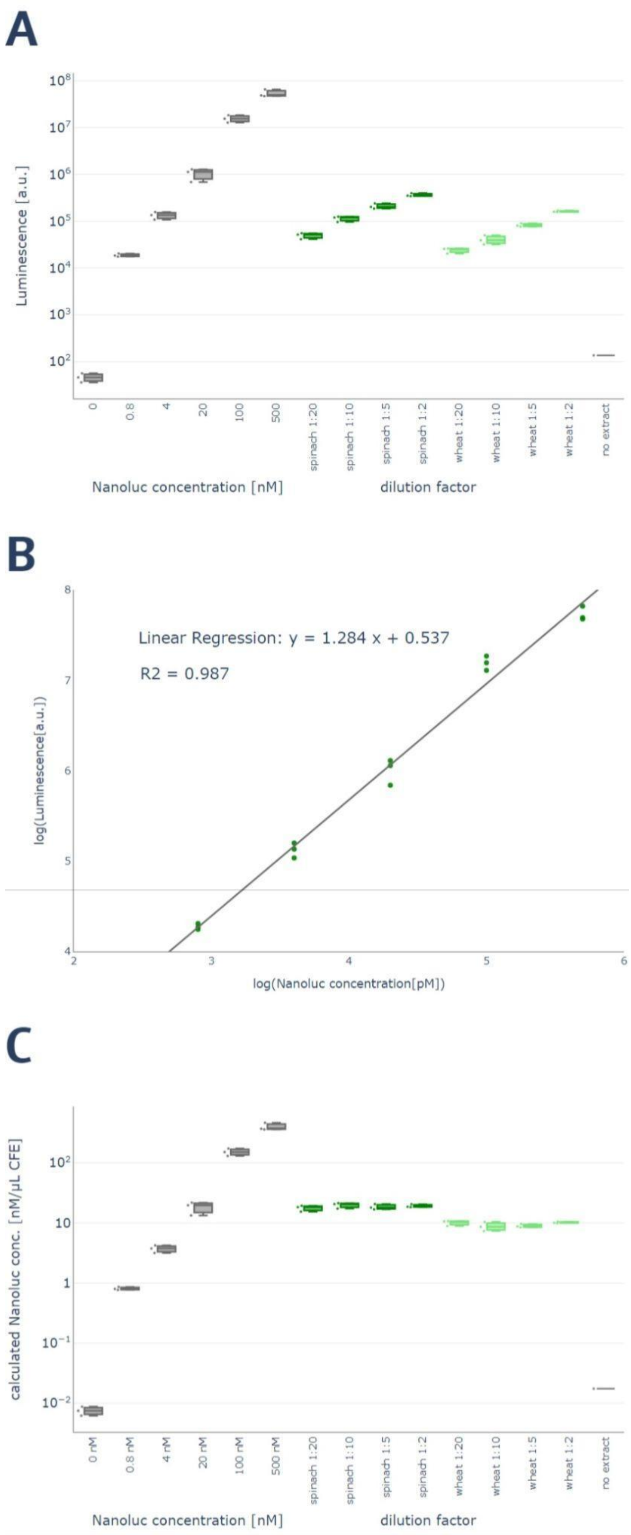

Figure S4: **Calibration of luminescence output of cell-free systems with purified NanoLuc.** (A) Raw luminescence values of purified NanoLuc (gray) and different dilutions of the spinach (dark green) and wheat (light green) cell-free systems after the synthesis reaction. (B) Calibration curve showing luminescence output as a function of NanoLuc protein concentration, including linear regression and R^2 value. (C) Calculated NanoLuc concentration per µl CFE reaction using data from the calibration curve in B, yielding 18.8 ± 1.8 nM for spinach and 9.5 ± 0.9 nM for wheat. Calculated total luminescence is very similar between dilution factors, indicating robust correction for absorption by the green extracts. Cell-free reactions were set up manually with a total volume of 10 µl and UTC 7.0 plasmid. Luminescence was measured after 4 hours of incubation at 20°C (N=3).

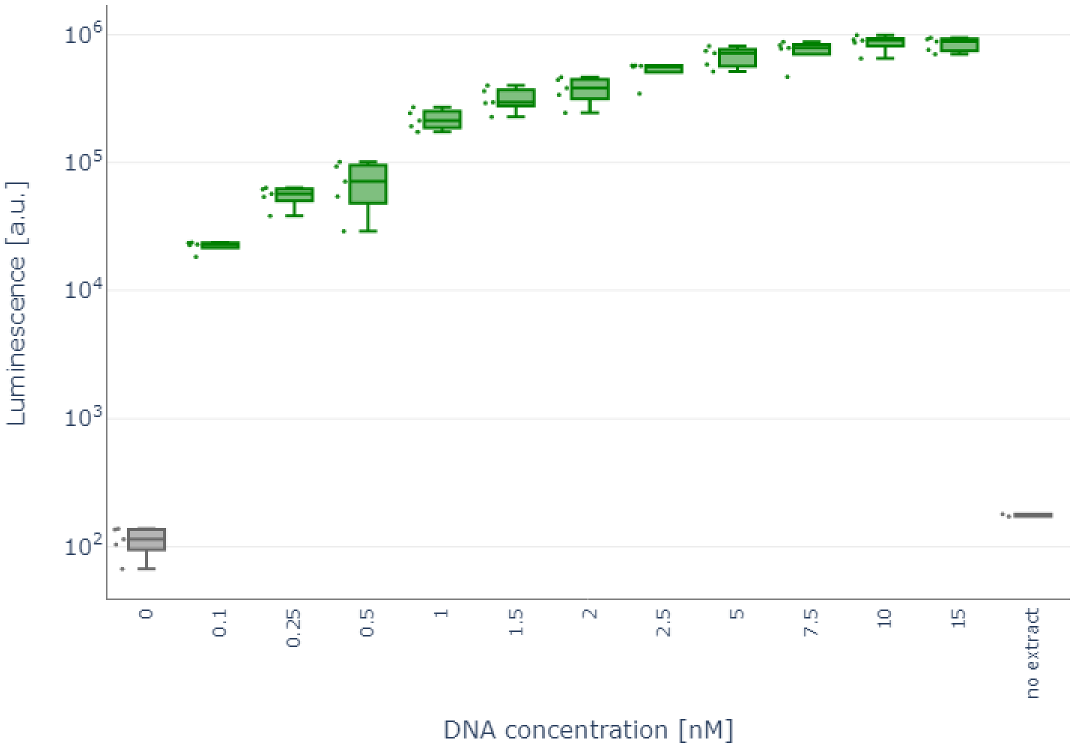

Figure S5: Effect of DNA concentration on cell-free protein production. NanoLuc luminescence signals obtained in CFE with varying template DNA concentrations. Highest expression is found at 10 nM UTC plasmid DNA concentration, all tested concentrations show expression above background. Negative controls either lack extract or DNA. Cell-free reactions were set up with a total volume of 2 µl and NanoLuc activity was measured after 4 hours of incubation at 20°C (N=5).

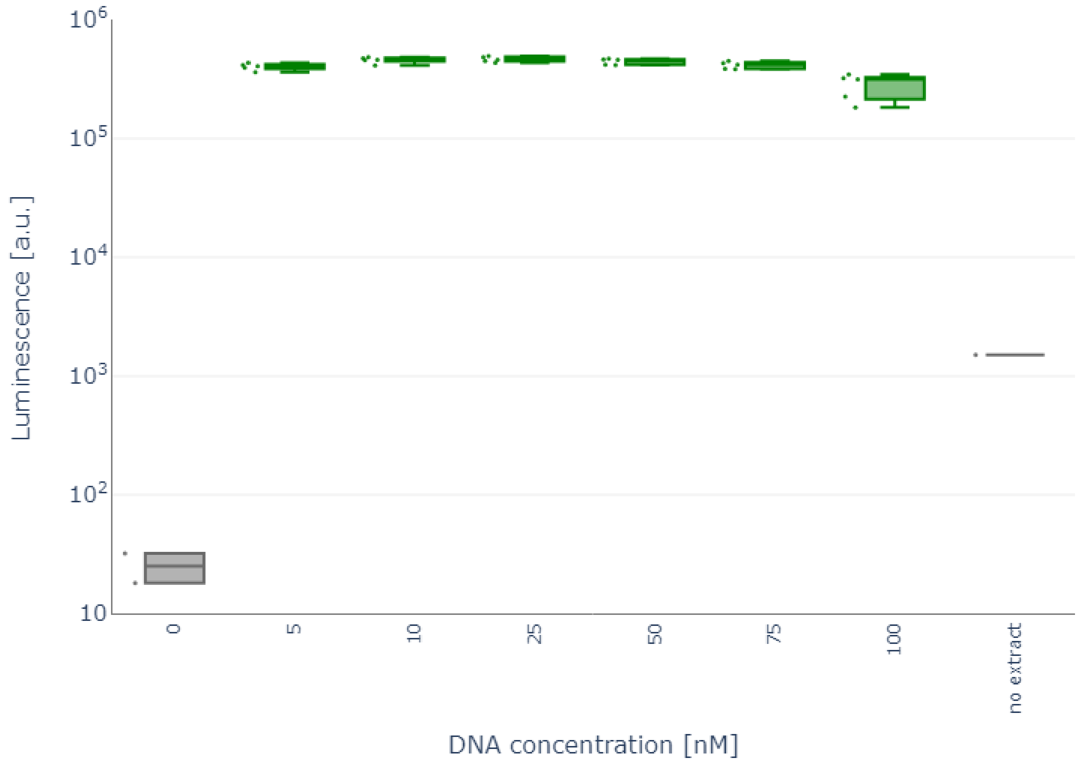

Figure S6: Effect of high DNA concentration on cell-free protein production. NanoLuc luminescence signals obtained in CFE with varying template DNA concentrations. Highest expression is found again at 10 nM UTC plasmid DNA concentration, all tested concentrations show expression above background. Negative controls either lack extract or DNA. Cell-free reactions were set up with a total volume of 2 µl and NanoLuc activity was measured after 4 hours of incubation at 20°C (N=5).

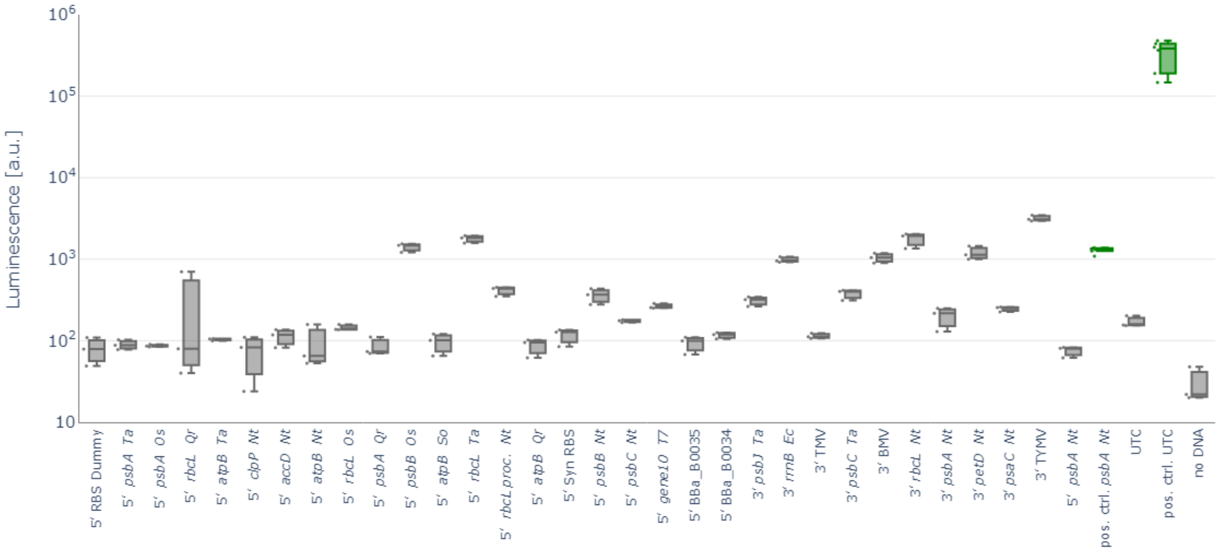

Figure S7: Background luminescence of template DNA plasmids in absence of cell-free extract. NanoLuc luminescence signals obtained in reactions lacking chloroplast extract. Negative control with included spinach extract lacks DNA. Positive controls containing all reagents were run alongside and measured at the same time. Mean expression was at least 17 times higher when extract was included. Reactions were set up with a total volume of 2 µl and NanoLuc activity was measured after 4 hours of incubation at 20°C (N=3).

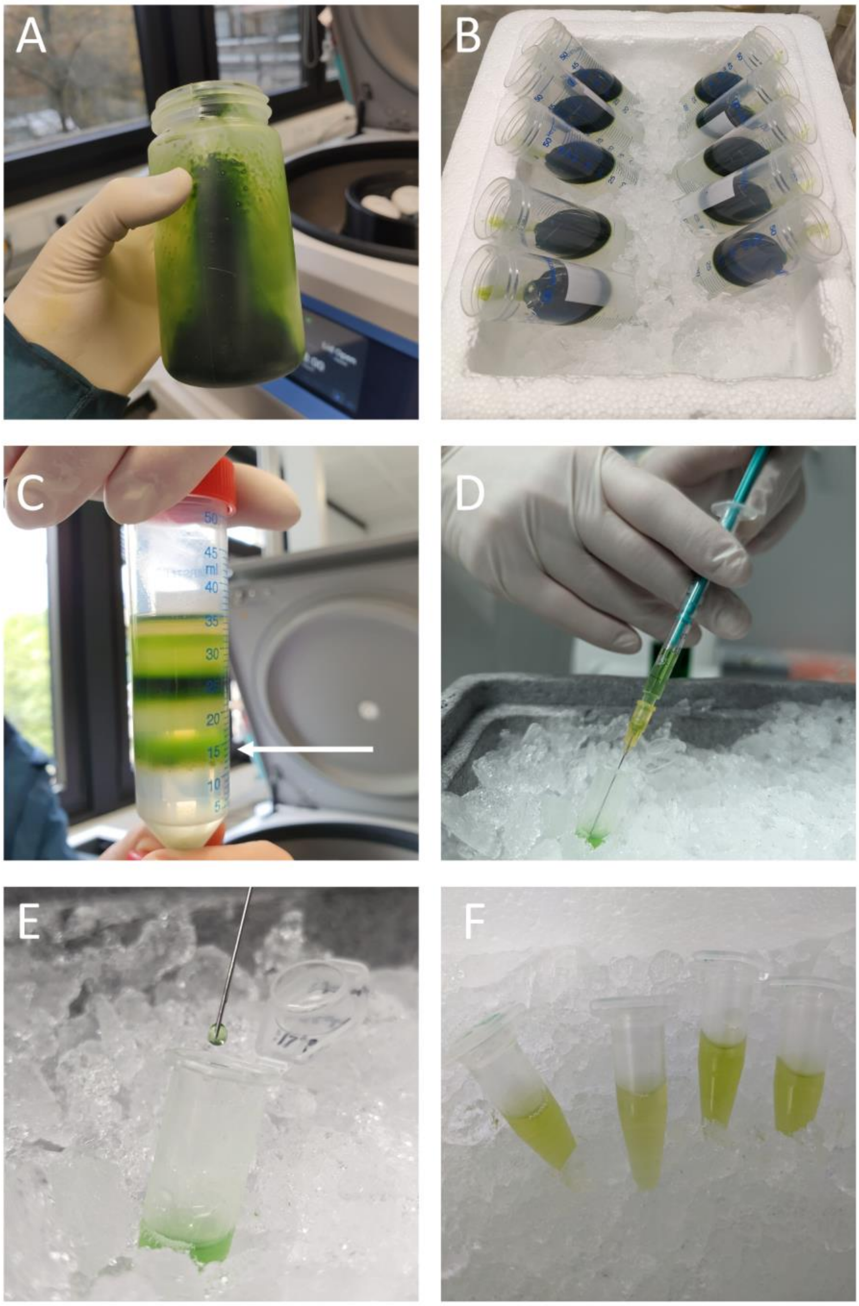

Figure S8: **Images of the chloroplast isolation workflow.** (A) Chloroplast pellet in centrifuge bottle after homogenization, filtering and first centrifugation. Importantly, very little starch is visible at this step. Starch, in high concentrations visible at this step as a white pellet, greatly reduces intact chloroplast yield. (B) Washed chloroplast suspension layered on top of Percoll step gradients. (C) Gradient after density centrifugation. White arrow indicates the position of intact chloroplasts. In layers above, broken chloroplasts and thylakoid membranes are visible. (D & E) Needle lysis of chloroplasts. During lysis (E), droplets are formed at the needle tip by applying gentle pressure on the needle plunger. (F) Final, functional cell-free extracts exhibit a slight green color.

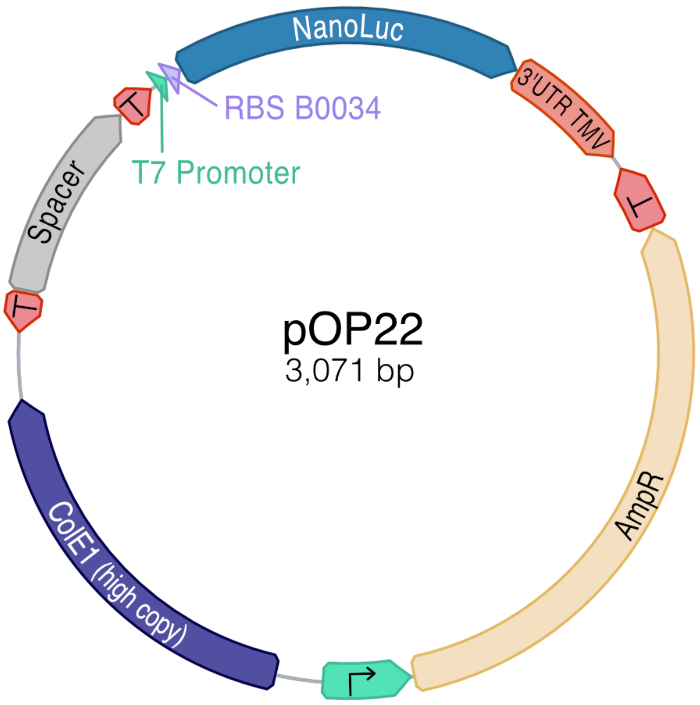

Figure S9: Plasmid map of the highest expressing DNA template for chloroplast cell-free expression The plasmid contains the T7 promoter, the RBS B00034 from the iGEM registry, serving as a 5'UTR, and the TMV 3'UTR from the tobacco mosaic virus. The NanoLuc coding region is codon optimized for the chloroplast genome of *Chlamydomonas reinhardtii*. The plasmid has an ampicillin resistance and a high-copy version of the pMB1 origin of replication, derived from the pUC vectors. The plasmid can be ordered and the full sequence can be found under the following link: https://www.addgene.org/216626/

Supplementary Tables

Table S1: Common methodological problems and suggested troubleshooting solutions.

Protocol step	Possible problems	Solutions
Chloroplast isolation buffer	Polyvinylpyrrolidone powder dissolves very slowly or clumps up	Prepare the buffers the day before the chloroplast isolation, add polyvinylpyrrolidone as the last reagent and stir in overnight in the cold room
	ß-Mercaptoethanol is not stable in solution	Add fresh just before chloroplast isolation
	Change in pH in HEPES and EDTA stock solutions	Measure pH of cold buffer solution before each isolation and adjust if necessary
Plant growth/ Plant material	Timing of harvest not ideal for the specific plant species	Age of the plant is important for successful preparation of active extract. It varies between plant species and may require individual fine-tuning
	Yield of the plant material is too low for chloroplast isolation	Increase number of plants or growth time, in general at least 150g of material is required for chloroplast isolation
	Starch content too high	Lower light levels during plant growth, extend dark incubation time, reduce fertilization
	General health of plants suboptimal (drought stress, light stress etc.)	Optimize growing conditions. Generally, plants are happier in the greenhouse than in a phytochamber with suboptimal spectral quality
	Plant material is too hard, preventing successful homogenization	Remove stems or midribs prior to homogenization, extend dark incubation period
	Detection of dirt after the first centrifugation	Wash leaves thoroughly
Dark incubation	Expression in CFE reaction too low due to senesced chloroplasts	Reduce dark incubation time and change plant growth conditions to reduce starch content
	Chloroplasts rupture too early during chloroplast isolation after leaves are removed from plant	Only remove the leaves from the plant immediately prior to homogenization, provide appropriate temperature, sufficient soil moisture levels and airflow during dark incubation
Homogen-isation	Too many ruptured chloroplasts after blending step	Reduce blending time
	Large, non-blended leaf pieces in homogenate	Pre-cut leaves into small pieces prior to blending with the help of scissors, ensure sufficient buffer volume is used, reduce the amount of plant material during blending and blend in multiple batches instead,

		remove leaf midribs
Filtration	Difficulties in creating sufficient hand pressure without bursting the cloth layers	Use very large pieces of cloth or process batch after batch
	Lint in chloroplast extract	Switch cheesecloth manufacturer, ensure miracloth is wrapped on the outside of cheesecloth
Initial centrifugation	Excessive starch visible as white part of the pellet after centrifugation	Increase dark incubation time
	Initial pellet is very thin	Re-Centrifuge decanted supernatant, fill centrifugation bottles to maximum possible volume, increase plant mass, optimize homogenization
Gradient centrifugation	No clear bands are distinguishable in the solution	Load less material on each gradient and split to more gradient tubes
	Unexpected band in highest percoll concentration step	Optimize gradient percoll concentrations according to specific density of chloroplasts
	Percoll is too expensive	Use sucrose
	Band of intact chloroplasts too thin	Use more plant material, reduce starch content of plant material, optimize plant material or blending steps, use fewer gradients (see above)
	Chloroplasts destroyed after removal from gradient centrifugation tube	Use wide-mouth serological pipettes for extraction of chloroplasts from gradient, pipette slowly
Chloroplast washing	Chloroplasts destroyed during washing	Use cut-off P1000 wide bore tips to transfer chloroplast suspensions to minimize shear forces
	Large losses between washing steps and very green supernatant	Increase centrifugation times
Chloroplast lysis	DTT & PMSF are not stable in lysis buffer, amino acids precipitate	Add fresh to lysis buffer aliquots, freeze and store as 1M DTT & 0.1M PMSF stock solutions, use fresh DTT and PMSF stocks, some amino acids are not soluble in water; Resuspend lysis buffer as much as possible immediately before use
	Insufficient lysis	Increase shear forces: higher number of needle passes, hold needle tip at the bottom of 1.5ml reaction tube during aspiration to create additional pressure. Hold needle over top of solution during dispersion and disperse slowly to create individual liquid droplets instead of a continuous stream

	Clear supernatant with low protein concentration and low expression after centrifugation	Optimize intactness of chloroplasts, lower starch concentration, handle chloroplasts gently during isolation to minimize shear forces. Working extracts usually have a slightly green color and protein concentrations above 25 mg/ml
Translation buffer preparation	Clumps / precipitate in translation buffer solution and amino acid stock solution	Some amino acids do not dissolve well in water, thoroughly mix both solutions immediately before use
	pH is not in the correct window	Carefully titrate the Hepes buffer to 7.3 using KOH. Adjust the pH of the final translation buffer to pH 7-8. Even a slightly diminished pH can result in inactive CFE reactions.
	Magnesium and potassium concentration might not be optimal	Magnesium and potassium concentrations may need to be titrated for any chloroplast cell-free system individually as the respective optimum might differ
Cell-free reaction/ Measurement	No reporter gene expression can be observed, without noticing one of the previous problems	Double-check the protein concentration by Nanodrop or BCA assay to check if the protein concentration of the extract is high enough. Typically, a working extract has a protein concentration of at least 25 mg/ml. Check if the reaction buffer is functional. Ensure plasmid DNA is pure and free of NanoLuc protein. Optimize magnesium & potassium concentration if creating an extract from a novel species.
	Measured expression levels using the same reagents drop significantly between measurements	Freeze cell-free extract, creatine kinase and translation buffer in liquid nitrogen and store at -80°C, use smaller aliquots
Constructs	High Luminescence from DNA solution alone	Heat-treat DNA solution to denature co-purified Nanoluc proteins (refer to methods section)
	No expression can be observed with DNA template of interest	Include a positive control with a DNA template, which has been shown to work in chloroplast cell-free extracts before, such as our universal test construct, which is available via Addgene. Some genetic parts behave very differently compared to findings from *in-vivo* studies, such as the *psbA* 5'UTR, which allows for very high expression *in vivo*, but shows low expression in chloroplast cell free extracts

Table S2 : Sequences of all regulatory elements used in this study.

**Type**	**Name**	**Sequence**	**length (bp)**	**Description**
Promoter	T7 promoter	TAATACGACTCACTATAG	18	core promoter region of the T7 promoter from *Escherichia* phage T7
Promoter	P*rrn16* Nt	GCTCCCCCGCCGTCGTTCAATG AGAATGGATAAGAGGCTCGTGG GATTGACGTGAGGGGGCAGGGA TGGCTATATTTCTGGGAGCGAAC TCCGGGCGAATACGAAGCGCTT GGATACAGTTGTAGGGAGGGAT TT	135	promoter of the plastidial 16S ribosomal RNA (*NitaCr105*) of *Nicotiana tabacum*
Promoter	P*rrn16* Cr	CAGGCAACAAATTTATTTATTGTC CCGTAAGGGGAAGGGGAAAACA ATTATTATTTTACTGCGGAGCAG CTTGTTATTAGAAATTTTTATTAA AAAAAAAATAAAAATTTGACAAAA AAAAATAAAAAAGTTAAATTAAAA ACACTGGGAATGTTCTAACAATC ATAAAAAAATCAAAAGGGTTTAA AATCCCGACAAAATTTAAACTTTA AAGAGT	217	promoter of the plastidial 16S ribosomal RNA (*CreCp.r003100.r RNA*) of *Chlamydomonas reinhardtii*
Promoter	P*rrn16* Ta	ATAAGAGGCTTGTGGGATTGAC GTGATAGGGTAGGGTTGGCTAT ACTGCTGGTGGCGAACTCCAGG CTA	69	promoter of the plastidial 16S ribosomal RNA (*TraeCr087*) of *Triticum aestivum*
Promoter	P*psbA* Nt	GATCTACATACACCTTGGTTGAC ACGAGTATATAAGTCATGTTATA CTGTTG	52	promoter of Photosystem II protein D1 (*psbA; NitaCp001)* of *Nicotiana tabacum*
Promoter	P*rbcL* Nt	GGGGGAAGTTCTTATTATTTAGG TTAGTCAGGTATTTCCATTTCAAA AAAAAAAAAAGTAAAAAAGAAAA ATTGGGTTGCGCTATATATATGA AAGAGTATACAATAATG	110	promoter of Ribulose bisphosphate carboxylase large subunit (*rbcL*; *NitaCp031*) of *Nicotiana tabacum*
5'UTR	*accD* 5'UTR Nt	AAGTGTTCCCCCAGATTCAGAAC TTTTTTTCAATACTCACAATCCTT ATTAGTTAATAATCCTAGTGATTG GATTTCTATGCTTAGTCTGATAG GAAATAAGATATTCAAATAAATAA	180	5'untranslated region of acetyl-CoA carboxylase beta subunit (*accD*; *NitaCp032*)

		TTTTATAGCGAATGACTATTCATC<br>TATTGTATTTTCATGCAAATAGG<br>GGGCAAGAAAACTCT		of *Nicotiana tabacum*
5’UTR	*atpB* 5’UTR Nt	CAAATGAAAGACTTTCTCAAGAT<br>TCTGATTCATCCACTTGAGATTTT<br>AAAATTAAAATAGGTTGGGTGGG<br>CTTGCAAATTCACTCAGTCTCAG<br>TGAATAAGTAAACAATTGAATCG<br>GTTCAATTGCATGGTGCCAACGA<br>AATCGAGTGCTAATTCCCATTTT<br>ATTGAATTAACCGATCGACGTGC<br>TAGCGGACATTTATTTTGAATTC<br>GATAATTTTTGCAAAAACATTTCG<br>ACATATTTATTTATTTTATTATT	255	5’untranslated region of ATP synthase subunit beta (*atpB*; *NitaCp030*) of *Nicotiana tabacum*
5’UTR	*atpB* 5’UTR Qr	CCTAGATGTGAAAATAGGAGGA<br>GTTCGCCCATGAAAAGCAAAGCA<br>TGAAACTAAAACTCTAAAACTAAA<br>GGGTATAGGTAAAAAAAAAATAG<br>GCTAGGCATAAATCGATAGGCTT<br>AAATATTAACTAAGAAATGAGATA<br>AGGGCACCAATAAGATAGAAAAA<br>ATGAATCGTAAATAGAAATAGAG<br>TTCCGGTTCGAATTCGATAAATA<br>ATATGGATGATATTGTCTATAATG<br>ATAGTCAAATGAAAGACTTTCTC<br>AAGACTTTTATTGATCCGCCTGA<br>GATTTTGAAAATGAGTTGGTTGA<br>ACTTGAAAATTAACTCATTGAAAT<br>TGAATAAATAAACAATCGAATTG<br>GATTCGATTGGATGGTACCAACG<br>AAATCTAGTGCAGTGCGAAACCC<br>CATTTATTATGGAATTATTATTGA<br>ATTAACCGATCAACTTGCTTTTCT<br>ATCGAACATTTTTTTTATTTCAAT<br>AATTTTCGCAAAAAAAAATTTCGA<br>CATATTATATTTTATT	506	5’untranslated region of ATP subunit beta (*atpB*; *HCS81_pgp061*) of *Quercus robur*
5’UTR	*atpB* 5’UTR So	GTGAAAATATGCAGAATTCTCTC<br>ATGAAAGGATAAAAGAATAGGCT<br>ACTCATAAATCTATATACTAAATC<br>GAAACTAAGTCCCAGTACGATAG<br>AAATAATGAATCATAAAAAAATAT<br>AGTTTTAGAGTTCGGGTTCGATT<br>TCCATAGATAATCTAGAAAGGAG<br>TGTCTATAATGATAGGCAAATAA<br>AAGACTTTCTCGGGATTTTTGGT<br>CATCCGTTTGATATTTTGAAAATA<br>GGCGGATTGCAATTTCAAATTGA<br>ATAGAAATAGAATAATTCAATTCC<br>AAAAAGTAAACAATTGAATTGGA<br>GTCCTTTTTTTTGCTGGTACCAA<br>CAAAATTTATTGCTAACCCCTATT	453	5’untranslated region of ATP subunit beta (*atpB*; *SpolCp032*) of *Spinacia oleracea*

		TCTTATTTAATTAATCGATCAGCT TGCTATCGGACATTTTTTTTATTT TGGATTCGATAATTTTCATTTTTG CAAAAAATTTCGACATACTTTACT ATATATT		
5’UTR	*atpB* 5’UTR Ta	TTTGTATATCGAAGTCCTAGATA GGAAAGTAGAGTAGGCACAGAT CCTCCACAAAAGGCAAAATGTAT ATGAAAAAAAGATTGATTGAACT TTCCAACGGACTCATTCCATGAG TAAACGATTGAATGGGATTCGCT TGGGCAACGAAATCAAGTCCTG GTCCCCTTTTCTCTCTTATTGAAT TAACTAATTCATTTCCTTTTTACT TTTGGATTTTTTTTGATTTGATTT GGCATTATTCAACAATAAAAAAA GAAAAATTTCGACAAATTCCTTTT TTTTAATTATGTGATAATT	297	5’untranslated region of ATP subunit beta (*atpB*; *TraeCp029*) of *Triticum aestivum*
5’UTR	*clpP* 5’UTR Nt	GTTTCCACCTCAAAGTGAAATAT AGTATTTAGTTCTTTCTTTCATTT A	48	5’untranslated region of ATP-dependent Clp protease proteolytic subunit (*clpP*;) of *Nicotiana tabacum*
5’UTR	*gene10* 5’UTR T7	GGCAGACCACAACGGTTTCCCA CTAGAAATAATTTTGTTTAACTTT AAGAAGGAGATATACAT	63	5’untranslated region of Major capsid protein (*gene10*; *T7p45*) of *Escherichia* phage T7
5’UTR	*psbA* 5’UTR Nt	AATAAAAAGCCTTCCATTTTCTAT TTTGATTTGTAGAAAACTAGTGT GCTTGGGAGTCCCTGATGATTAA ATAAACCAAGATTTTACC	88	5’untranslated region of Photosystem II protein D1 (*psbA*; *NitaCp001*) of *Nicotiana tabacum*
5’UTR	*psbA* 5’UTR Os	TAACAAGCCTTCTATTATCTTTCT AGTTAATACGTGTGCTTGGGAGT CCTTGCAATTTGAATAAACCAAG ATCTTACC	78	5’untranslated region of Photosystem II protein D1 (*psbA*; *AKK66_gp001*) of *Oryza sativa*
5’UTR	*psbA* 5’UTR Qr	TAACAAGCCCTCAATTATCTATTT CTATTTATAGAGAATCGTGTGCT TGGGAGTCCCTGATGATTAATGA TTAAATAAACCAAGATTTTACC	92	5’untranslated region of Photosystem II protein D1 (*psbA*; *HCS81_pgp089*)

				of *Quercus robur*
5'UTR	*psbA* 5'UTR So	TAACAATCTTTCAATTTCTATTTCTAGCGAATTTGTGTGCTTGGGAGTCCCTGATGATTAAATTAAATAAACCAAGATTTTACC	84	5'untranslated region of Photosystem II protein D1 (*psbA*; *SpolCp002*) of *Spinacia oleracea*
5'UTR	*psbA* 5'UTR Ta	TAACAAGCCTCCTATTATCTATATTCTAGTTAATACGTGTGCTTGGGAGTCCTTGCAATTTGAATAAACCAAGATCTTACC	81	5'untranslated region of Photosystem II protein D1 (*psbA*; *TraeCp001*) of *Triticum aestivum*
5'UTR	*psbB* 5'UTR Nt	GCTTCTCTTTGTTCCTACGAACAGAATTGTTCCATTATTACCAACAGAATAGAACACCCTTGTTCGGAAATAATCGACTGAACAAGAGTGGTCCATAGGATAGTCATATTATAGTCTTTTCCAATGCAATAAAGTTACGTAGTGTCTATTTATCTTTGATATAAGGGGTATTTCC	175	5'untranslated region of Photosystem II CP47 reaction center protein (*psbB*; *NitaCp052*) of *Nicotiana tabacum*
5'UTR	*psbB* 5'UTR Os	GTATAGAATAGATCTGCTTCTCTTTCTTCTTACGAACAGAATTGGCTTCTTATTTTTAATGGAATGAAATAAATATTCACGCTTTCTGACACAGAATCCCCTAGAAGGGTTAGGTACATAGGATATGGATAGTCTTTGCCAATGCGATAAAATAAAGTGACATCGTGTCTATTTTTCTTTGCTAAAGGGGTATTTCC	197	5'untranslated region of Photosystem II CP47 reaction center protein (*psbB*; *AKK66_gp055*) of *Oryza sativa*
5'UTR	*psbC* 5'UTR Nt	GTTATTTGTACCAGTAACCGGTTTATGGATGAGTGCTCTTGGAGTAGTCGGTCTAGCCCTGAACCTACGTGCCTATGACTTCGTTTCTCAGGAAATTCGCGCAGCGGAAGATCCTGAATTTGAGACTTTCTACACCAAAAATATTCTCTTAAACGAAGGTATTCGCGCTTGGATGGCGGCTCAAGATCAGCCTCATGAAAACCTTATATTCCCTGAGGAGGTTCTACCAC	230	5'untranslated region of Photosystem II CP43 reaction center protein (*psbC*; *NitaCp016*) of *Nicotiana tabacum*
5'UTR	*rbcL* 5'UTR Os	GGATTTGGTGAATCAAATCCATGGTTTAATAACGAAGCATGTTAACTTACCATAACAACAACTCAATTCTTATCGAATTCCTATAGTAGAATTCCTATAGCATAGAATGTACACAGGGTGTACCCATTATATATGAATGAAACATATTATATGAATGAAACATA	325	5'untranslated region of Ribulose bisphosphate carboxylase large subunit (*rbcL*; *AKK66_gp073*) of *Oryza sativa*

		TTCATTAACTTAAGCATGCCCCC CATTTTCTTTAATGAGTTGATATT AATTGAATATCTTTTTTTTAAGAT TTTTGCAAAGGTTTCATTTACGC CTAATCCATATCGAGTAGACCCT GTCGTTGTGAGAATTCTTAATTC ATGAGTTGTAGGGAGGGACGT		
5'UTR	*rbcL* 5'UTR Qr	GTATTTGGCGAATCAAATATCAT GGTCTAATAACGAACCATTCTAA TTAGTTGATAATTTTTTGAAAGGA TTCCTTGAAAGGTTTCATTAACTC CTAATTCATGTCGAGTAGACCTT GTTGTTGCGAAAATTCTTAATTC	140	5'untranslated region of Ribulose bisphosphate carboxylase large subunit (*rbcL*; *HCS81_pgp060*) of *Quercus robur*
5'UTR	*rbcL* 5'UTR Ta	GGATTTGGTAAATCAAATCCATG GTTTAATAACGAACCGTGTTAAC TTACCATAACAACAACTCAATTC CTATCGAATTCCTATAGTGGAAT TCCTATAGGATAGAACATACACA GGGTGTACGCATTATATATGAAT GAAACATATTCATTAACCTAAGC ATGCCCTCAATTTTCTTTAATGAG TTGATATTATATTAATTGAATATC CTTTTTGTTTTACGAGATTTTTGC TAAAGTTTCATTTACGCCTAATTA ACATCGAGTAGACCCTGTTATTG TGAGAATTCTTAATTCAAGAGTTA TAGGGAGGGACTT	317	5'untranslated region of Ribulose bisphosphate carboxylase large subunit (*rbcL*; *TraeCp030*) of *Triticum aestivum*
5'UTR	*rbcL* 5'UTR So	AAATACATGGTCTATTAACGAAC CATTTTGATTAGTTGATAATATTA ATTGAGAATTTGATGAAAGATTG CTATAAAAGGTTTCATTAAGGCC TAATTTATGTCGAGTAGACCTTG TTGCTTTGTTGTAAAAATTAAAAT TTGAAGTTGTAGGGAGGGACTT	176	5'untranslated region of Ribulose bisphosphate carboxylase large subunit (*rbcL*; *SpolCp033*) of *Spinacia oleracea*
5'UTR	*rbcL* 5'UTR processed Nt	GTCGAGTAGACCTTGTTGTTGTG AGAATTCTTAATTCATGAGTTGTA GGGAGGGATTT	58	processed version of 5'untranslated region of Ribulose bisphosphate carboxylase large subunit (*rbcL*; *NitaCp031*) of *Nicotiana tabacum*
RBS	BBa_B0034	AGAGAAAGAGGAGAAATAATC	21	RBS B0034 of the Community RBS part collection
RBS	BBa_B0035	AGAGATTAAAGAGGAGAATAATC	23	RBS B0035 of the Community RBS

				part collection
RBS	Synthetic RBS	AAATTCGATAGAGATGAAATTGGAGCTCTAGAGAATTCAGTTGTAGGGAGGGATCC	56	Synthetic RBS based on the 5'untranslated region of *rbcL* (*NitaCp031*) of *Nicotiana tabacum*
RBS	RBS_Dummy	AGAGTGTCAGGATACCCGATAATC	24	5'untranslated region with low translational activity
CDS	NanoLuc	GTTTTCACTTTAGAAGACTTCGTAGGTGACTGGCGTCAAACAGCAGGTTATAATTTAGACCAAGTTTTAGAACAAGGTGGTGTATCAAGTTTATTCCAAAATTTAGGTGTTTCTGTTACTCCAATTCAACGTATCGTATTAAGTGGTGAAAATGGTCTTAAAATTGACATCCATGTTATTATTCCTTATGAAGGTCTTTCAGGTGACCAAATGGGTCAAATTGAAAAAATTTTTAAAGTAGTTTATCCTGTAGATGACCATCACTTTAAAGTAATTTTACACTATGGTACTTTAGTAATTGATGGCGTTACACCTAATATGATTGACTACTTTGGTCGTCCTTATGAAGGTATTGCTGTTTTTGATGGTAAAAAATCACAGTTACAGGTACATTATGGAATGGTAATAAAATTATTGACGAACGTTTAATCAATCCTGATGGTTCATTATTATTCCGTGTTACAATTAATGGTGTTACAGGTTGGCGTCTTTGTGAACGTATCCTTGCT	510	Coding sequence of the NanoLuc luciferase
3'UTR	*petD* 3'UTR Nt	AAATTTTTAAAGATTCAATTGTGAAATAACACGACATGTGTATCTAGGGAATAGTTTCTTCAAAGCGAATTCTCCCTAGATACATCTATTCAATTTAATTCTGAATTTATTTTGAATATATGATATATTAATATATTAATTGTGCTAAAGAGTTTCAATCTATTTTCACTAAGTAAGTCCAATAGAT	187	3'untranslated region of Cytochrome b6-f complex subunit 4 (*petD*; *NitaCp051*) of *Nicotiana tabacum*
3'UTR	*rpoA* 3'UTR Nt	AAATCTATTGGACTTACTTAGTGAAAATAGATTGAAACTCTTTAGCACAATTAATATATTAATATATCATATATTCAAAATAAATTCAGAATTAAATTGAATAGATGTATCTAGGGAGAATTCGCTTTGAAGAAACTATTCCCTAGATACACATGTCGTGTTATTTCACAATTGAATCAATTTAAAA	189	3'untranslated region of DNA-directed RNA polymerase subunit alpha (*rpoA*; *NitaCp057*) of *Nicotiana tabacum*

		AT		
3’UTR	*psaC* 3’UTR Nt	AATGATACGTTCGAGAAAACTCTACTTGAATCCATTTAATTTTTTTTACCGACAAACCTGTGCTCGAAAATCACAATATTTTGAGCACGGGTTTTTATG	99	3’untranslated region of Photosystem I iron-sulfur center (*psaC*; *NitaCp084*) of *Nicotiana tabacum*
3’UTR	*psbA* 3’UTR Nt	AATCCTGGCCTAGTCTATAGGAGGTTTTGAAAAGAAAGGAGCAATAATCATTTTCTTGTTCTATCAAGAGGGTGCTATTGCTCCTTTCTTTTTTT	95	3’untranslated region of Photosystem II protein D1 (*psbA*; *NitaCp001*) of *Nicotiana tabacum*
3’UTR	*psbC* 3’UTR Ta	AAGATTTTCTTATTTATACCTGTTCTACTTTTTTTCTGTTCTGGCTCGGTTATTCCATCTAGCCGAGCCATTCATTCCTTTTTATGAAAGAAAGATAAGGGACAGAAAAAAAAAAAAAA	119	3’untranslated region of Photosystem II CP43 reaction center protein (*psbC*; *TraeCp007*) of *Triticum aestivum*
3’UTR	*psbJ* 3’UTR Ta	AATAATCGGAGGGACCAGATTGTAAACATGAAAAAGTAGGAGCTTAGCGGGTCCTTACCCCCCTTTATCTGATTAGAGCGGAAAGGACCCGCGGAATTTTTACTCTTATAACGCGAATTGATTCTATTCGATTCACTCTTATGAAGCAACAAGAAAAAGAGATCACTCGAGGATCCAATATCTTATTCCACGAAGGAAGTATCCTGGAAATCCTTGATTTAGTTTCGAGTAATAAACTAATAAAACCTTAATCAAAACTATTCAACTAGCCTAAAAAATAAAAAAAAA	288	3’untranslated region of Photosystem II reaction center protein J (*psbJ*; *TraeCp036*) of *Triticum aestivum*
3’UTR	*rbcL* 3’UTR Nt	AAAAACAGTAGACATTAGCAGATAAATTAGCAGGAAATAAAGAAGGATAAGGAGAAAGAACTCAAGTAATTATCCTTCGTTCTCTTAATTGAATTGCAATTAAACTCGGCCCAATCTTTTACTAAAAGGATTGAGCCGAATA	142	3’untranslated region of Ribulose bisphosphate carboxylase large subunit (*rbcL*; *NitaCp031*) of *Nicotiana tabacum*
3’UTR	*rrnB* 3’UTR Ec	AAGTAGAAACGCAAAAAGGCCATCCGTCAGGATGGCCTTCTGCTTAATTTGATGCCTGGCAGTTTATGGCGGGCGTCCTGCCCGCCACCCTCCGGGCCGTTGCTTCGCAACGTTCAAATCCGCTCCCGGCGGATTT	222	3’untranslated region of the ribosomal RNA operon copy B (*rrnB*; *b3971*) of *Escherichia coli*

		GTCCTACTCAGGAGAGCGTTCA CCGACAAACAACAGATAAAACGA AAGGCCCAGTCTTTCGACTGAG CCTTTCGTTTTATTTGATG		
3'UTR	TMV 3'UTR	AAATAATAAATAACGGATTGTGT CCGTAATCACACGTGGTGCGTA CGATAACGCATAGTGTTTTTCCC TCCACTTAAATCGAAGGGTTGTG TCTTGGATCGCGCGGGTCAAAT GTATATGGTTCATATACATCCGC AGGCACGTAATAAAGCGAGGGG TTCGAATCCCCCCGTTACCCCCG GTAGGGGCCCA	192	3'untranslated region of Capsid protein (*TMVgp6*) of Tobacco Mosaic Virus
3'UTR	TYMV 3'UTR	AAGTTCTCGATCTTTAAAATCGTT AGCTCGCCAGTTAGCGAGGTCT GTCCCCACACGACAGATAATCG GGTGCAACTCCCGCCCCTTTTCC GAGGGTCATCGGAACC	107	3'untranslated region of Coat protein (*TYMVgp3*)
3'UTR	BMV 3'UTR	AAGGTGCCTTTGAGAGTCTACTT TTGCTCTCTTCGGAAGAACCCTT AGGGGTTCGTGCATGGGCTTGC ATAGCAAGTCTAGATGCGGGTAC CGTACAGTGTTGAAAAACACTGT AAATCTCTAAAAGAAACCA	133	conserved 3'untranslated region of all Brome Mosaic Virus RNAs (1,2 and 3)

Table S3: Translation buffer components and stock solutions.

Reagent for translation buffer	Stock concentration [mM]	Final concentration in translation buffer [mM]	Stock volume per µl reaction [µl]
HEPES	2000	15	0.008
KOAc	3500	60	0.017
MgOAc	3000	10	0.003
NH_4OAc	2900	30	0.01
ATP	500	2	0.004
GTP	100	1	0.01
CTP	100	1	0.01
UTP	100	1	0.01
Creatine Phosphate	1000	8	0.04
20 amino acids (each)	50	2	0.008
DTT	1000	5	0.005
Spermidine	100	0.1	0.001

Table S4: Reaction components and stock solutions.

Reagent for reaction mastermix	Stock concentration	Final concentration in CFE reaction	Stock volume per µl reaction [µl]
Translation buffer	-	13% v/v	0.13
Creatine kinase	7.5 U/µl	0.025 U/µl	0.033
T7 Polymerase	20 U/µl	0.28 U/µl	0.014
PEG 3350	20% w/v	2% w/v	0.1
RNase Inhibitor	40 U/µl	0.5 U/µl	0.013
Plasmid Template	50 nM	10 nM	0.2
